# Housing With Care for Older People: A Scoping Review Using the CASP Assessment Tool to Inform Optimal Design

**DOI:** 10.1177/19375867221113359

**Published:** 2022-08-22

**Authors:** Suyee Jung, Lesley Uttley, Junjie Huang

**Affiliations:** 1 School of Architecture, University of Sheffield, United Kingdom; 2 School of Health and Related Research, University of Sheffield, United Kingdom

**Keywords:** housing for older people, housing with care, extra care housing, evidence-based design, conceptual framework, scoping review

## Abstract

**Objective:**

The purpose of this scoping review is to synthesize and map available evidence on the design of “housing with care” (HWC) schemes to inform design decisions built on objective data from previous research, which is key to ensuring such schemes are fit for purpose for older people.

**Background:**

HWC is becoming increasingly recognized as a model for developing housing schemes for older people and balances independent living with elevated levels of care. However, as this scheme is still relatively novel, there are currently no established theoretical frameworks to inform design.

**Methods:**

Scoping review, thematic analysis, and mapping methods were used to comprehensively search for and synthesize evidence that links design with assessments of quality-of-life data for HWC schemes. Study findings for each included paper were subject to data extraction for inductive analysis, and the quality of each study was assessed using a modified critical appaisal skills programme (CASP) checklist.

**Results:**

Our searches yielded 821 unique references, of which 18 unique articles met the inclusion criteria. The outcomes of interest were the design considerations or features in HWC schemes and their impact on the residents. The main themes identified were related to design element, accessibility, maneuverability, views, design procedure, and quality of life (QOL). Further subthemes identified across papers were identified to create a comprehensive map of the key features to consider in designing HWC schemes.

**Conclusion:**

This review provides an initial framework for designers and architects to (1) understand the effect of each design element of HWC and (2) inform design to ultimately improve the QOL of aged people.

## Introduction

The proportion of people aged 65 and over is growing faster than any other group worldwide, accounts for over 9% of the world total population, and is expected to increase to 16% by 2050 (United Nations Department of Economic and Social Affairs Population Division, 2019). The transition from independence to increased reliance on care is an important turning point in the human life cycle and is likely to accompany a decrease in physical and mental health. Studies show that the built environment plays a particularly important role in reducing disability and improving well-being (Barbara & Barnartt, 2014; Fancourt & Finn, 2019; Roelofsen, 2014) and underscore the need to broaden studies on healthcare environments to encompass long-term care environments (e.g., assisted living [AL] facilities, retirement homes) within more rigorous research frameworks (Ulrich et al., 2010). The terms *built environment* and *physical environment* are often used interchangeably. Built environment usually refers to land use planning, street connectivity, and transportation (Woolf & Aron, 2013) and may include housing, green spaces, safety, and sanitation (Salgado et al., 2020). Here, references to the physical environment include both housing structures and their immediate surroundings.

Local governments are encouraged to create settings and enforce standards for newly built house and home modifications suited to an aging population. Likewise, as the proportion of older people increases, so does the need to both provide care in healthcare facilities and to create homes that provide care (Mazuch, 2017). Housing with care (HWC)—a subcategory of AL that is viewed as an alternative care environment model—is a housing model geared toward aging populations in which design is centered on functionality and aims to integrate housing with the adequate and accessible spaces and care services offered. HWC is becoming an increasingly important part of long-term care systems (Chapin et al., 2001) and aims to provide an age-friendly physical environment that is integrated with care service, so residents benefit from increased independence and quality of life (QOL). A residential environment for older people that combines housing with a range of care services is currently considered to be the optimum model (Regnier & Denton, 2009). However, although it has been established that the design of a given space substantially affects a person’s behavior in their environment, methodological flaws have obscured attempts to collect “objective, evaluative, and discrete” data on optimal design features for physical environments in AL settings (Cutler, 2000, 2007). In stark contrast to nursing homes, guidelines for designing the physical environment are not standardized for AL settings (Cutler, 2007) and are nonexistent for HWC settings.

The realm of research on housing design for older people is bound by certain limitations, such as the difficulty of conducting randomized control trials and the selection of objective data. Despite these limitations, primary evidence is strengthened when research findings are replicated and reproduced using the scientific method, so they can be acknowledged as credible evidence (Peavey & Vander, 2017). Hence, a rigorous review is needed to comprehensively assess how the literature could support the design and assessment of future HWC approaches and to provide an initial framework for designers. However, studies that have assessed the literature to establish a consensus on the most important principles to guide HWC design and objective criteria for designing HWC environments are completely lacking.

### Research Aims and Objectives

Therefore, the present study aims to (1) comprehensively review research on residences for older people and (2) to assess the quality of this evidence. The overall objective of this review is to investigate and assess the existing evidence on the housing environment for older people in relevant literature published over the last 15 years to provide an initial framework for designers of HWC facilities.

## Method

We performed a scoping review of the literature and synthesis using thematic analysis—a method of analyzing qualitative data to identify, analyze, and report patterns contained the data set (Braun & Clarke, 2006; Thomas & Harden, 2008). A scoping review determines the extent of the existing literature in a given field and can be used as a research tool to map existing literature on a certain topic. This review follows Preferred Reporting Items for Systematic Reviews and Meta-Analyses (PRISMA) guidelines (Moher et al., 2009).

### Information Sources and Search Strategy

**Figure 1.**
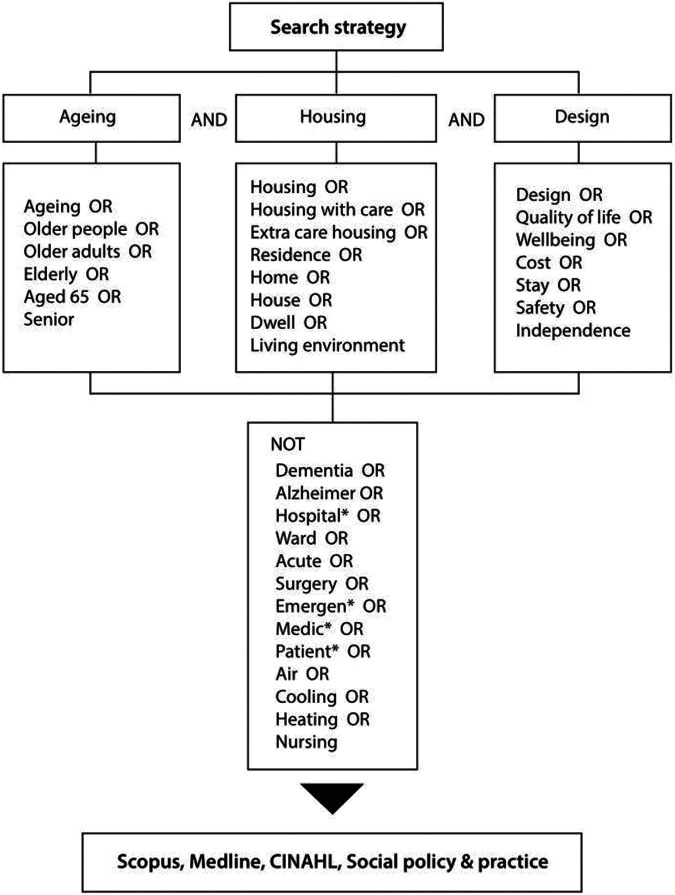
Study search strategy.

A search strategy was developed in conjunction with advice from an information specialist. Key word searches of article titles and abstracts were conducted using three conceptual categories (Online Appendix 1): (1) living environments (housing, extra care housing, housing with care, residen*, home, house, dwell*, living environment), (2) aging (older people, elderly, older adults, aged 65, ag$ing, senior), and (3) design (design, cost, quality of life, well$being, stay*, safety, independen*). As this study focuses on socio-psychological factors in designing residential environments for older people, we intentionally excluded terms that apply to people with serious sensory or cognitive impairment and environmental factors (e.g., Dementia, Alzheimer, Hospital*, Ward, Acute, Surgery, Emergen*, Medic*, Patient*, Air, Cooling, Heating, and Nursing; Figure 1).

Electronic searches were performed using four databases from November 5, 2019 to January 8, 2020, using Scopus, Medline with Web of Science, CINAHL, and Social Policy & Practice with Ovid. Test searches for the sensitivity and specificity of research terms were conducted with corresponding adjustments. Medical subject headings (MeSH) were included, Scopus and Medline were replaced by Web of Science and PubMed, and Social Policy & Practice was added according to the advice of a university information specialist. While the aims and theoretical rationale for HWC is documented in the literature from around 2001, the evidential literature to examine such schemes that have already been built has not appeared until much more recently. Additionally given that interest in housing for older populations has grown exponentially over the last few years, and this review’s focused specifically on HWC rather than care homes for the elderly in general, the decision was made to restrict the literature searches to studies conducted within 15 years prior to the search date (January 2005 to December 2019). MeSH searches were conducted to include all the available studies in the search results.

### Eligibility Criteria

Studies were eligible for inclusion if they met the following criteria: published (1) in English, (2) in the last 15 years, and (3) in peer-reviewed academic journals. Research that did not focus on architectural design was excluded. No limit on geographical region or participant ethnicity was imposed to allow a variety of cultures and populations to be reviewed. Definitions of “older” vary with context therefore this review focused on studies of people aged 65 and older, given that it is at the upper end of the global median retirement age. Inclusion criteria were as follows: age selection criteria (people aged ≥ 65) and articles dealing with architectural design items (e.g., house modification, housing type, physical barriers). The exclusion criteria were as follows: social care policy, environmental design (e.g., heat, energy, air quality), nursing (≥65 and under medical care), articles regarding potential population transfer to the status of receiving care, and people aged ≥ 65 living in institutional settings.

### Study Selection

All retrieved references were imported into the Mendeley, and duplicates were removed. All study titles and abstracts were assessed against the review eligibility criteria by one reviewer (S.J.) in the first phase of screening. In the next stage, remaining studies were retrieved for full-text assessment. A proportion (20%) of all records generated through searches were independently screened by a second reviewer (L.U.).

### Data Collection Process and Data Items

The final selection of articles was parsed to extract information relating to each study’s aims, abstracts, sample size and methods, variables, and dependents and were tabulated in Microsoft Excel by one reviewer (S.J.). Themes and subthemes were derived based on the inclusion of subordinate concepts and classified as variables and outcomes to create a second data table for use in thematic analysis mapping. A proportion (30%) of all data extracted were independently screened for accuracy by a third reviewer (J.H.).

### Quality Assessment and Applicability

CASP was used to review the quality of the research included in the present study, as it allows for a systematic assessment of trustworthiness and quality of various study designs. Adopting tools from this compendium of checklists facilitates quality assessments across different study designs. The quality and applicability of each study was assessed using modified CASP checklists by one reviewer (S.J.). To better adapt the checklists to this research question, three extra questions were added: (1) demographical applicability (Online Appendix 2), (2) architectural design focus (Online Appendix 3), and (3) design applicability to HWC (Online Appendix 4). A proportion (30%) of all quality assessments were independently screened for accuracy by a third reviewer (J.H.).

### Thematic Synthesis Mapping

A synthesis of studies was conducted using thematic analysis to inform a conceptual model of HWC encompassing all types of study designs (Thomas & Harden, 2008) using an iterative and inductive approach to analyzing qualitative research across a variety of epistemologies and research questions. A translation table was created from all relevant themes extracted from each paper using Microsoft Excel. First-order structures were defined by taking concepts and recognizing the same concepts from each study, although not expressed using identical words. Relevant themes were classified into variables and outcomes, grouped by similar topics, and subgrouped as second-order structures. Concepts were then mapped using Microsoft Visio to visualize the relationships between themes. Cross-comparisons resulted in original third-order structures (i.e., maps) to inform the new conceptual framework.

## Results

### Study Selection, Designs, and Characteristics

**Figure 2.**
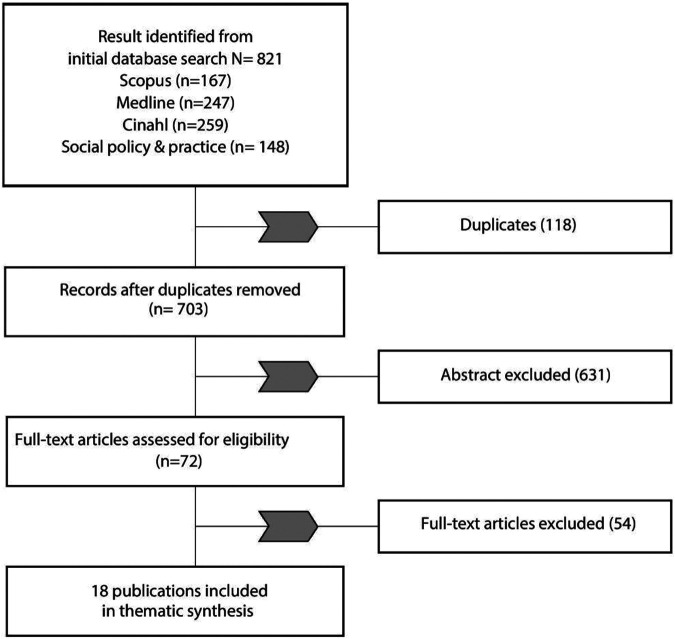
Preferred Reporting Items for Systematic Reviews and Meta-Analyses flow diagram of study selection and exclusion.

Of the 821 citations returned in our initial searches (167 from Scopus, 247 from Medline, 259 from CINAHL, and 148 from Social Policy & Practice), 18 articles that focus on the relationship between aging, housing, and QOL were included in the thematic analysis. The process of study identification and selection is summarized in the PRISMA diagram in Figure 2.

Details of the characteristics for each study and sample are provided in Table 1. A total of 3,694 participants (3,686 people aged ≥ 65 and eight caregivers) were represented in the included studies, with sample sizes ranging from seven to 1,188. One case study included four different schemes but did not report the number of individual participants. The ages of older people included in the samples ranged from 52 to 98 years. Most studies included mixed genders, while one study (Chin & Quine, 2012) was 100% female. The selected studies were conducted in nine countries (four in the United States, four in Australia, three in Sweden, two in the United Kingdom, one in the Netherlands, one in Chile, one in Finland, one in Belgium, and one in Iran). One study (Shin, 2018) was conducted in the United States but targeted a specific ethnicity within a multiethnicity societal setting.

**Table 1.** Details of the Reviewed Articles.

	Author (Year)	Age	Sample Size (*n*)	Country	Duration of the Study	Methodological Approach	Housing Context	Variables	Measures
1	Rodiek and Fried (2005)	62–99 (avg. 83.97)	133	United States	Single time point	Cross-sectional method	Long-term care facilities	Edited photos depicting environmental conditions (e.g., benches on existing walkway, white metal awning for comfort, opening to an area beyond, alternative type of fencing for views, trees instead of bar chips and lamppost for greenery, paths in landscape and windows)	Residents’ preferred visual images
2	Burton and Sheehan (2010)	Avg. 85	80	United Kingdom	Single time point	Qualitative method	Care homes	Situation of care homes, authority of providers, style of building. Size of the care homes, sex, and physical condition of the residents	Importance of design of their homes, satisfaction with home, walking around home, preferred layout, access, corridor design, location, and garden design
3	Currin et al. (2012)	65–80	63	Australia	Six months	Qualitative method	Traditional homes	Age, comorbidities, a number of falls, urinary incontinence, level of depression (K–10), EQ-5D, Cognition (Abbreviated Mental Test Score), and Frenchay Activities Index	Recommendations that were implemented
4	Chin and Quine (2012)	Over 65	36 (100% female)	Australia	23 months	Qualitative method	Traditional homes and residential aged care facilities	Older environment of older women (own home + aged care facilities)	Quality of life concerns and fears of losing privacy and friendship in aged care facilities
5	Pizzi et al. (2013)	72 ± 6	40	Chile	Single time point	Mixed method Cross-sectional Observation + Interview	State provided senior housing	Senior state housings	Physical barriers and risks for basic activities for daily living
6	Orrell et al. (2013)	N.R.	163	United Kingdom.	Six months	Cross-sectional method	Extra care housing	Quality of extra care schemes (EVOLVE), dependency of the participants	Quality of life on CASP-19
7	Kylén et al. (2014)	67–70	371	Sweden	Single time point	Qualitative method	Traditional homes	Age, sex, marital status, level of education type of housing, type of housing (one family house/rented or owned apartment in multifamily building), objective health, and functional limitations. Activities in daily life, independence, dependence on mobility devices, and depressive symptoms	Number of environmental barriers
8	Carnemolla and Bridge (2014)	52–96	89	Australia	Single time point	Mixed method approach (quantitative and qualitative analyses)	Traditional homes	Utility score before and after home modification	Quality of life dimensions in – independent living – mental health – relationships – senses
9	Smith et al. (2016)	Avg.78.7	1,188	United States	15 months	Observational cohort	Traditional homes	Clinical and functional domain (RAI-HC), community accessibility, and mixed land use	Frequency of outdoor mobility
10	Pirinen (2016)	N.A.	N.R.	Finland	Single time point	Qualitative comparative analysis	Communal senior housing	Producer-driven (“for the elderly”) and a resident-driven (“by the elderly”) housing	Target group, immaterial promise or benefit, strategy for delivering the promise, role of the residents, relationship of the concept to architecture, initiator, source of innovation, and external references
11	Nakhodaeezadeh et al. (2017)	60–92	128	Iran	Single time point	Cross-sectional questionnaire	Traditional homes	Living environment (EVOLVE), quality of life (CASP-19), control, autonomy, self-realization, and pleasure	Perceived social support (MSPSS)
12	Van Steenwinkel et al. (2017)	63–84	Seven (and eight caregivers)	Belgium	Single time point	Qualitative case study	Residential care facilities	Physical and cognitive capacities, residential care environments	Experience of residents and caregivers, role of architectural features
13	Gobbens and Van Assen (2018)	65 and older (avg. 73.4)	1,031	Netherlands	Single time point	Cross-sectional questionnaire	Traditional homes	Environment factors (housing, facilities, nuisance, residents, neighborhood, stench, noise, and traffic)	Quality of life (physical, psychological, social, and environmental) nuisance had the strongest correlation with residents, traffic, and stench/noise
14	Shin (2018)	61–94 (avg. 77.8)	138	United States (Korean ethnicity)	Single time point	Mixed method (qualitative and cross-sectional)	Traditional homes	Unit layout, unit entrance, building shell and layout, building siting, living room, kitchen, bathroom, and bedroom	Individual features of the housing, experiential attributes of the building (e.g., thermal comfort, visual pleasure)
15	Lindahl et al. (2018)	67–94 (avg. 83)	28	Sweden	Single time point	Qualitative method	Extra care housing	Four different ECH settings	Sense of safety
16	Kim and Portillo (2018)	71–98	88	United States	Five months	Mixed method Case control	Senior living community (retirement community)	Two case-controlled buildings (high fall rate/low fall rate), age, and mobility	Environmental hazards (WeHSA)
17	Berglund-Snodgrass and Nord (2019)	60–95	18	Sweden	Single time point	Qualitative method	Extra care housing	Two different extra care housings in different spatial and environmental situations within a geographical boundary.	Space-time trajectories of safety-accessing, continuing, and reconstituting
18	Carnemolla and Bridge (2019)	Avg. 72	157	Australia	Single time point	Before and after cross-sectional questionnaire	Traditional homes	Type and location of home modification, and type of care	Care needs

*Note*. N.A.: Not applicable; N.R.: Not recognized; CASP-19: Quality of life scale; WeSHA; Westmead home safety.

The 18 included studies were performed in eight conventional homes (which were modified at the request of or to meet the requirements of the residents), eight residential care facilities including extra care housing (ECH), two care homes, and two communal senior housing facilities. The results of the selected studies and discussions of evidence found therein were grouped under the following major themes: A. Design Element; B. Accessibility; C. Maneuverability; D. View; E. Design Procedure; and F. Quality of Life.

### Quality Assessment

The results of the quality assessment utilizing the modified CASP checklist are summarized in Table 2 for qualitative studies, Table 3(a) for cohort studies, and Table 3(b) for case-control studies.

**Table 2.** Quality Appraisal of Retained Qualitative Research Publications.

Author (Year)	1	2	3	4	5	6	7	8	9	10	11	12	13	Overall Appraisal
Burton and Sheehan (2010)	√	√	√	√	√	√	?^a^	√	√	It provides a model for further user-centered research on design and well-being at all scale of the built environment	√	×	√	SAT
Chin and Quine (2012)	√	?	?	√	?	?	√	?	√	Suggestion of elements of building design and the makeup of the social environment potentially need further exploration to alter the experiences of the residents	√	√	√	SAT
Kylén et al. (2014)	√	√	√	√	√	√	√	√	√	Finding can be transferred to other Western countries that favor community-based healthcare and social services	√	×	√	SAT
Carnemolla and Bridge (2014)	√	√	√	√	√	×	?	?	√	Basis of an evaluation model that recognizes both physical role and well-being to capture the r benefits of home modification to deliver	√	√	√	SAT
Van Steenwinkel et al. (2017)	√	√	√	?	√	?	?	√	√	Suggesting design strategies for residential care facilities which enhances freedom	√	×	√	SAT
Shin (2018)	√	√	√	√	√	?	?	?	√	Comprehensive understanding of general environmental need and situation of an ethic group.	√	×	√	SAT
Lindahl et al. (2018)	√	√	√	√	√	?	√	√	√	Applicable to the design of extra care housing (ECH) for a sense of security	√	?	√	SAT
Berglund-Snodgrass and Nord (2019)	√	√	√	√	√	?	√	√	√	Contribution to an uncertainty of what qualifies for in terms of care and social life, and what residents expect and demand in ECH	√	×	√	SAT
Carnemolla and Bridge (2019)	√	√	√	√	√	√	√	?	√	Demonstrating the role of physical home design that contributes independent life	√	×	√	SAT

*Note*. Response options: yes √; no ×; and unclear ?. KP = key paper; SAT = satisfactory; FF = fatally flawed.

^a^ This question marked “unclear” if no formal ethical approval reported but no ethical concerns identified.

**Table 3.** Quality Appraisal of Retained Cohort Research and Case Control Research Publications.

Author, (Year)	1	2	3	4	5^a^	5^b^	6^a^	6^b^	7	8	9	10	11	12	13	14	15	Overall Appraisal
(a) Cohort Research																		
Rodiek and Fried (2005)	√	√	√	√	?	?	n/a	n/a	Hypothetical preferred features (rest facility, views, greenery, open transition, and walkways) of outdoor environment were substantiated	Precise	√	√	√	Preferred environment features in hypothetical and practice-based literature are supported by digitally modified image method	√	√	√	SAT
Pizzi et al. (2013)	√	?	√	√	n/a	√	n/a	n/a	State housing design is significant in basic, activities of daily living (BADLs) performance, limiting functionality, which is concerned demanding reaching requirements associated with height, extended to other inadequacies in design, or lack of elements, which act as barriers or bring potential risks	Precise	√	√	√	BADLs can increase functionality, by adapting height and adequate design	√	√	√	SAT
Orrell et al. (2013)	√	√	√	√	n/a	√	n/a	n/a	Elements of design related to accessibility, safety, working care, and security are associated with quality of life (QOL)	?	√	×	?	Universal needs as choice and control and personal realization can be promoted by better design of housing with care	√	√	√	SAT
Kylén et al. (2014)	√	√	n/a	√	×	√	n/a	n/a	Hundred percent of the home assessed had barriers; height/inaccessible position, low position at entrances, use requires hands, and low position in hygiene area	?	√	√	√	Quantitative assessments of aspects of home and health in different phases of the aging process	√	√	√	SAT
Smith et al. (2016)	√	?	n/a	√	√	√	n/a	n/a	Walkable, barrier-free sidewalks, access to public transportation, and decaying front porch or unstable front stairs deteriorate outdoor mobility	?	√	√	√	Housing barriers and community accessibility merit attention compensating older people’s declining health status and functional limitations	√	×	×	SAT
Nakhodaeezadeh et al. (2017)	√	√	√	√	n/a	√	n/a	n/a	Women and elders living in the flat-type houses, people living in big homes, and having guest rooms had higher level of social support	?	√	×	×	Applying simple standard tools for reforming housing design, educating architects about elder-friendly interior design, and implementing home modifications to support the needs of the elderly population	√	√	√	SAT
Gobbens and Van Assen (2018)	√	?	n/a	√	√	√	n/a	n/a	Housing, residents, and nuisance influence QOL in older adults. Home modification including smart home technology may make it more suitable	Precise	√	√	?	Environmental scales can be improved by removing nuisances	√	×	×	SAT
Shin (2018)	√	?	n/a	√	√	√	n/a	n/a	Allocate adequate space for bed, furniture, circulation space, and closet for two occupants in planning and specify well-organized shelving and hanging systems within the closet are recommended for bedroom design	?	√	√	×	The guideline is extensive as many critical issues are related to basic human needs along with needs for meaningful socialization and activities which can serve as the first step to planners and designers	×	√	×	SAT
Carnemolla and Bridge (2019)	√	√	√	√	×	×	n/a	n/a	Home modification significantly reduced formal care	?	√	√	√	Home modification directly support needing care and reduce amount of care required in the home	√	×	×	SAT
(b) Case Control Research																		
Kim and Portillo (2018)	√	√	√	√	√	n/a	n/a	n/a	n/a	√	√	√	√	√	√	√		SAT

*Note*. n/a = not applicable; KP = key paper; SAT = satisfactory; and FF = fatally flawed.

^a^ Response options: yes √; no ×; and unclear ?.

^b^ This question marked “unclear” if no formal ethical approval reported but no ethical concerns identified.

While reflexivity was nearly absent, seven of 18 studies included a statement placing the researcher culturally or theoretically (Burton & Sheehan, 2010; Gobbens & van Assen, 2018; Kim & Portillo, 2018; Nakhodaeezadeh et al., 2017; Orrell et al., 2013; Shin, 2018; Smith et al., 2016). Only two studies included an acknowledgment of the influence of the researcher on the research (Burton & Sheehan, 2010; Rodiek & Fried, 2005). Despite this, all 18 studies were deemed to satisfy the quality assessment CASP checklists.

### Data Extraction and Synthesis

**Figure 3.**
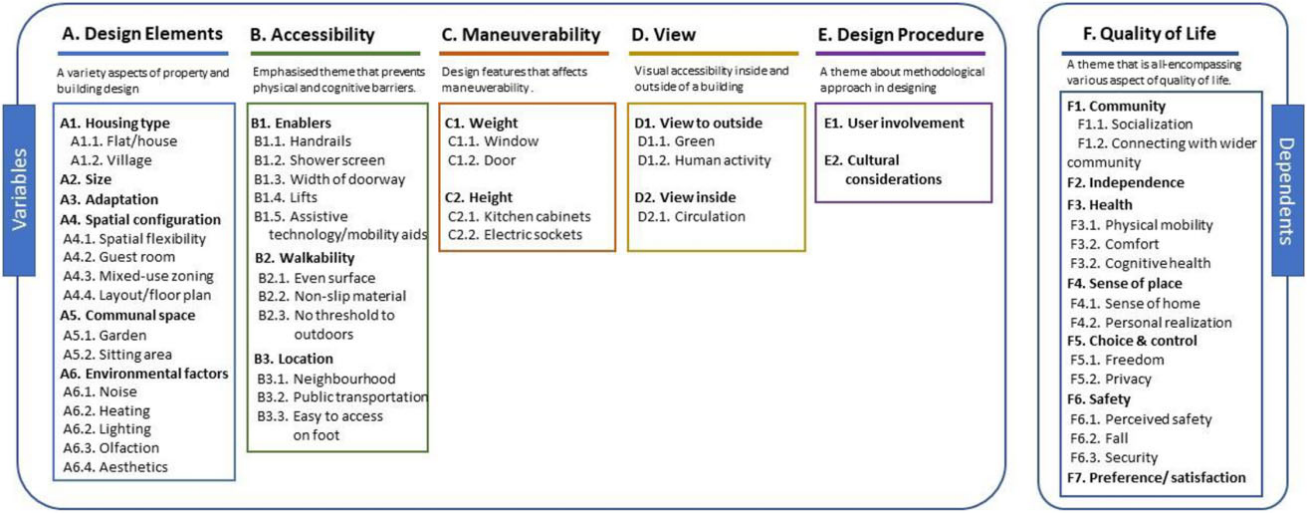
Thematic analysis translation.

Themes and subthemes were grouped into interventional design factors and their relevant effects on users. To address the need for objective data regarding physical design elements, themes were created to help elucidate the objective elements of the physical environment that contribute to the subjective dimensions of QOL, as well as the concept that HWC design can attribute to well-being in later life. Figure 3 shows the structures of the relevant main domains, themes, and subthemes.

### Thematic Mapping Variables

#### Theme A: Design elements

**Figure 4.**
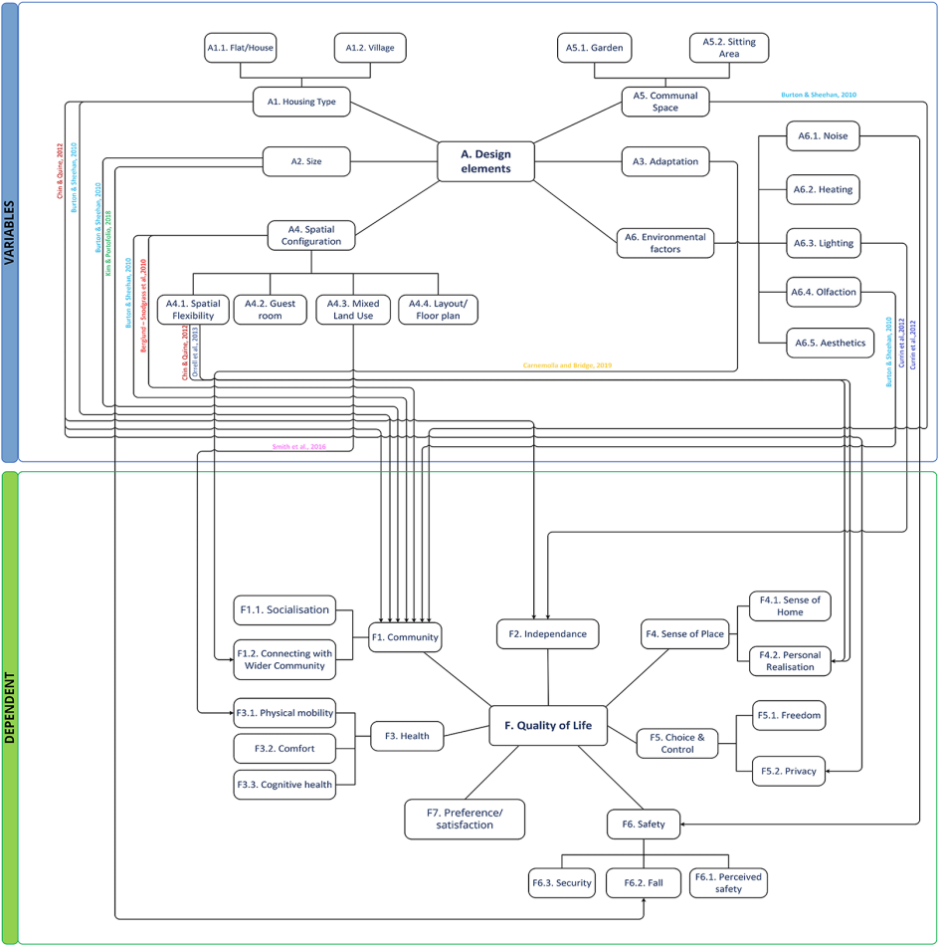
Thematic conceptual diagram between Group A and dependent groups.

This group represents thematic areas in the schemes and aspect of building design (Figure 4). Housing type is one factor related to the extent of user control (Pirinen, 2016). Some studies argued that room size (Burton & Sheehan, 2010) and the scheme size (Orrell et al., 2013; e.g., the number of living units) are associated with residents’ QOL. Orrell et al. (2013) stated that the size of a scheme is a factor in the relationship between building design and residents’ QOL owing to universal needs such as comfort, control, personal realization, and dignity; however, the authors admit that there may also be other variables that are difficult to measure. Although modification usually applies to traditional housing, the specific relationships between factors and outcomes are notable. *Environmental factors* are comprised of noise, heating, lighting, olfaction, and aesthetics. Shin (2018) stated that residents’ daily activities were affected by thermal, olfactory, and auditory comfort by the manner, operation, or adjustment of ventilation. These factors were described as universal needs across cultural divides and affected residents’ physical health and well-being, security, and fall hazard (Burton & Sheehan, 2010; Gobbens & van Assen, 2018; Kim & Portillo, 2018; Shin, 2018).

Aesthetics of buildings and decor may impact satisfaction (Burton & Sheehan, 2010; Orrell et al., 2013). Pirinen (2016) cited differences between housing designed for older adults as prospective residents and housing designed by older adults either having a designer or artist background or who were interested in social living. *Space configuration* is associated with socialization, feelings of happiness, sense of place, and home-likeness via flexible space, guest room, and layout (Berglund-Snodgrass & Nord, 2019; Burton & Sheehan, 2010; Chin & Quine, 2012; Kim & Portillo, 2018; Nakhodaeezadeh et al., 2017; Orrell et al., 2013; Shin, 2018; Van Steenwinkel et al., 2017). Spatial flexibility was identified in the user-driven design (Pirinen, 2016). Specifically, several studies state that spatial flexibility is linked to socialization and privacy (Berglund-Snodgrass & Nord, 2019; Burton & Sheehan, 2010; Shin, 2018). Unit entrance configuration was related to several issues: accessibility, usability, and security (Shin, 2018). Shin (2018) also recommended smooth transitions from corridor to doors for wheelchairs and ample storage for outdoor items and easier cleaning. *Size*—including both small-scale and generous spaces—influences feelings of freedom, social contact, and accessibility (Van Steenwinkel et al., 2017), while the lack of space triggered reduced usability owing to mobility and maneuverability issues, increased fall risk, and decreased socialization (Berglund-Snodgrass & Nord, 2019; Kim & Portillo, 2018; Shin, 2018). For example, residents in buildings with high fall rates reported a lack of space for mobility and maneuverability (Kim & Portillo, 2018). *Modification*: Currin et al. (2012) and Carnemolla and Bridge (2014, 2019) focused on home modification for older adults receiving care at home, while Pirinen (2016) emphasizes defining AL in terms of readiness for modification. Currin et al. (2012) indicates that the level of performance uptake of home modification recommendations was dependent on the combination of service availability and residents’ personal factors such as comorbidities. Kylén et al. (2014) found that housing adaptation compensates for the external control belief that older people can control their home environment by counterbalancing deteriorating functional capacity. Kim and Portillo (2018) and Carnemolla and Bridge (2019) validate the notion that home modification fosters independence through decreased care need. *Communal space* was one of the most frequently cited themes and presents increased opportunities for socialization (Berglund-Snodgrass & Nord, 2019; Lindahl et al., 2018; Orrell et al., 2013). The design features of communal space that affect variability of use and general satisfaction are flexibility and size.

#### Theme B: Accessibility

**Figure 5.**
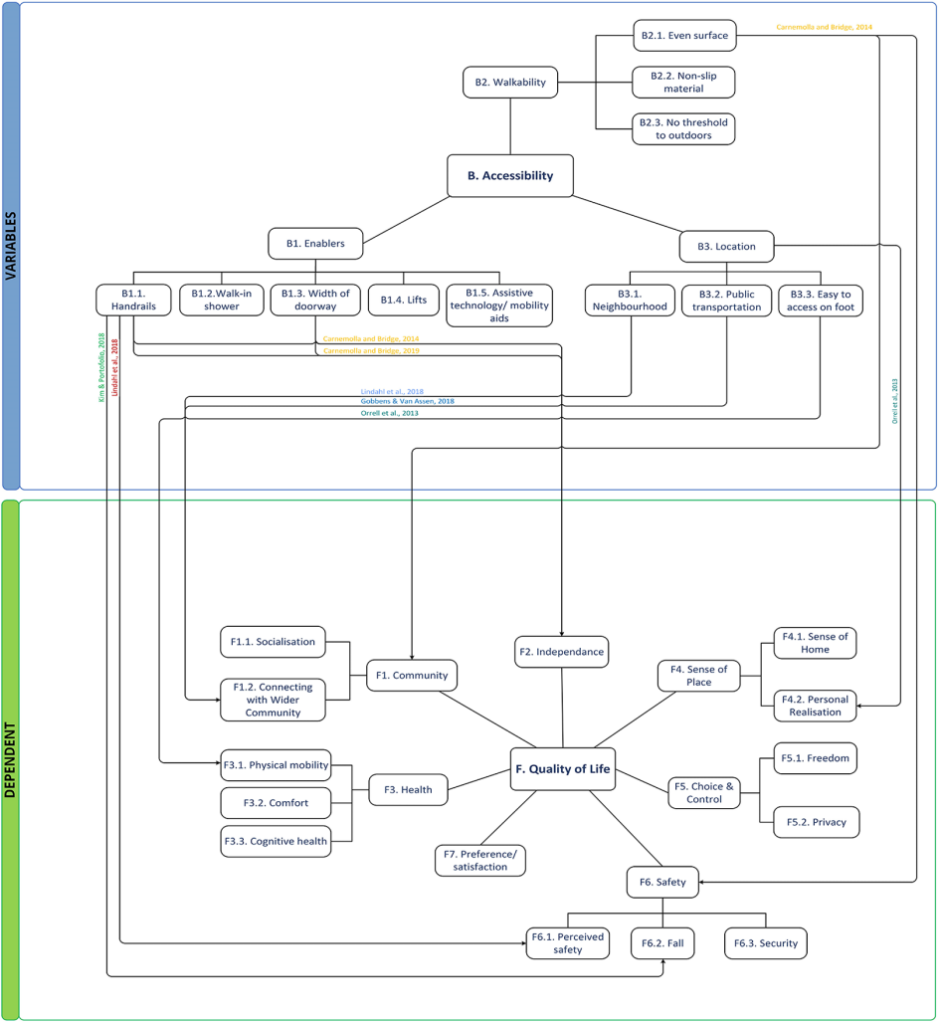
Thematic conceptual diagram between Group B and dependent groups.

Accessibility was emphasized to identify physical and cognitive barriers in building areas (Figure 5). Ten studies reported on *enablers*, *walkability*, and *location* (Berglund-Snodgrass & Nord, 2019; Burton & Sheehan, 2010; Carnemolla & Bridge, 2019; Currin et al., 2012; Gobbens & Van Assen, 2018; Kim & Portillo, 2018; Kylén et al., 2014; Nakhodaeezadeh et al., 2017; Pizzi et al., 2013; Shin, 2018; Smith et al., 2016). The subtheme *Enablers* consists of handrails, walk-in shower, widths of doorway, lifts, and assistive technology/mobility aids, where the main areas of focus in the literature are the bathroom and kitchen (Carnemolla & Bridge, 2019; Currin et al., 2012; Kim & Portillo, 2018; Kylén et al., 2014; Pizzi et al., 2013; Shin, 2018). Design features in hygienic areas include grabrail, handheld shower, shower screen, and commode area. The kitchen, bedroom, and entrance were associated with accessibility issues. Notably, Kim and Portillo (2018) focused on environmental safety related to fall hazards involving narrow width and lack of handrails. Kylén et al. (2014) stated that perceived functional independence can be measured through a Housing-Related Control Beliefs (HCB) Questionnaire; however, the data had low internal consistency. *Walking surface* concerns originate from surface, door slip, and doorsill unevenness, which are associated with both accessibility and safety (Orrell et al., 2013). This theme includes the quality of the sidewalk and entry barriers of the immediate exterior environment. Kylén et al. (2014) found that barriers and irregular walking surfaces are prevalent in the entrance environment. Removal of doorsills and nonslip treads on stairs are recommended (Currin et al., 2012). Stair unevenness has been found to be one of the main architectural barriers hindering the performance of basic daily activities in Chile’s senior state housings (Pizzi et al., 2013).

#### Theme C: Maneuverability

**Figure 6.**
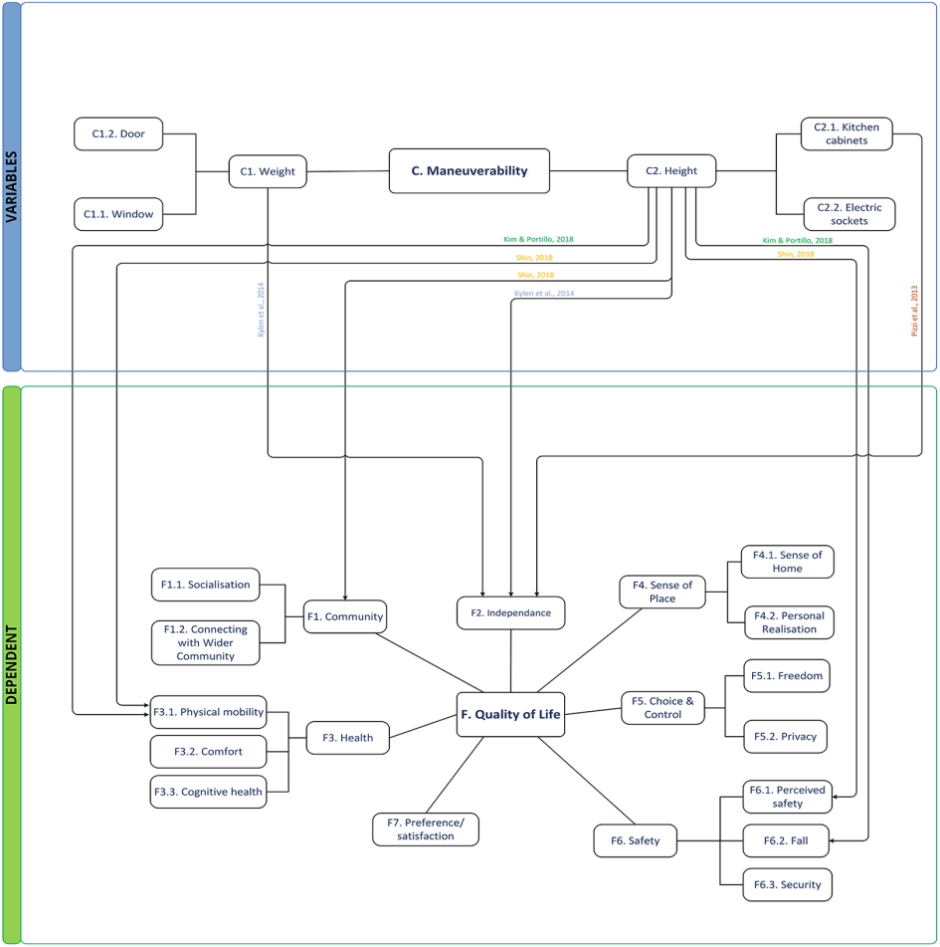
Thematic conceptual diagram between Group C and dependent groups.

*Maneuverability* involves features that could affect ease of use, such as the weight and height of doors and windows (Figure 6). Pizzi et al. (2013) identified that inadequate heights of essential elements such as cabinets and electrical outlets affected QOL. Walkability encouraged older people to go out independently and offered opportunities to exercise. The context of the scheme influences residents’ perceived security and connection with both the wider community the place. Finally, *garden* was mentioned as providing some small choices of spaces and activities to the female residents that were the focus (Chin and Quine, 2012). In addition, there was attention raised in designing barrier-free gardens for mobility (Shin, 2018). Shin (2018) suggested adequate space allocation should be given to the building site to allow social gathering, parking, and gardening behavior.

#### Theme D: View

**Figure 7.**
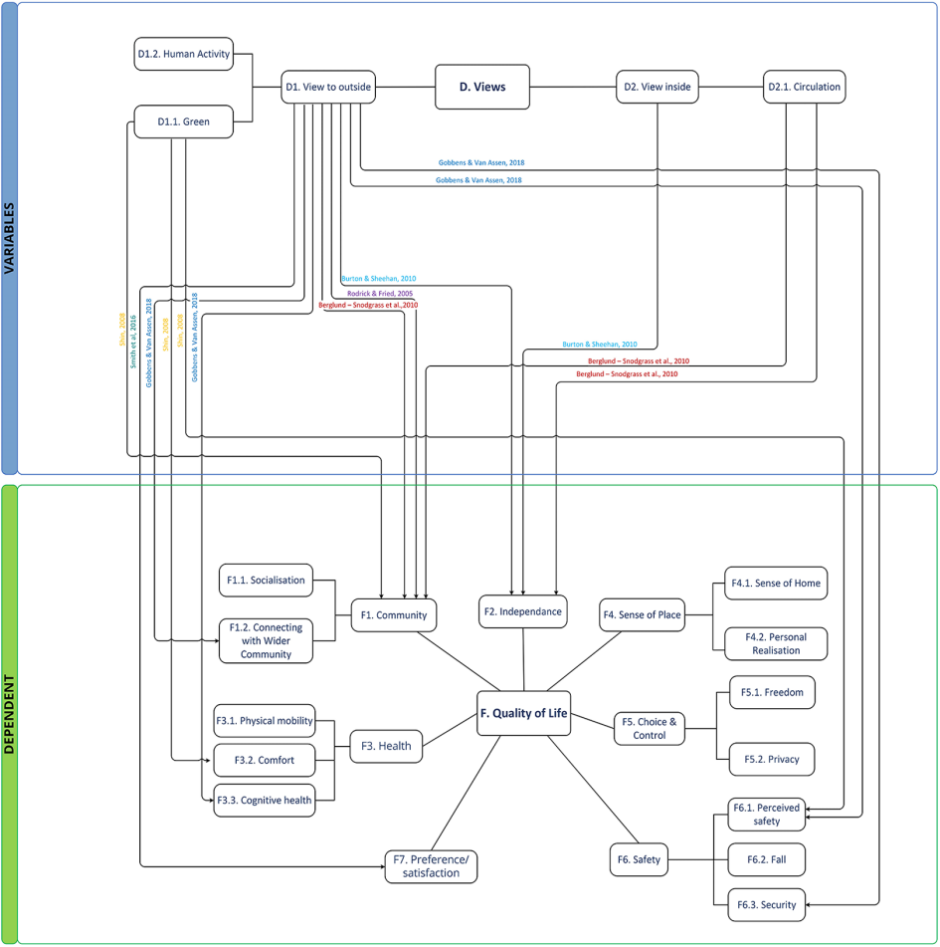
Thematic conceptual diagram between Group D and dependent groups.

This theme includes the subthemes *view to outside* and *view inside*. Rodiek and Fried (2005) verified the hypothetical preference of the view for green using a photographic comparison method. Outdoor view is largely associated with activity, perceived safety, and connecting with wider community, while views inside a building—including visibility of circulation—are associated with sense of control and community (Figure 7). Smith et al. (2016) reported that older adults in the care environment preferred more views, greenery, windows, and paths. Burton and Sheehan (2010) confirmed that immediate views from windows are more appreciated than location. Visual openness is highly correlated with perceived accessibility. *Views inside* result in an “open” and “friendly” atmosphere and provide the possibility of seeing more areas and facilitating movement in the space. Visual openness indoors can be achieved via an open floor plan or glass (paneled) walls and doors. Views of people coming and going in a communal area is linked to sense of community and choice of socialization. Burton and Sheehan (2010) represent it as a lighter, welcoming, less intimidating environment, where it is possible to see parts of the home and identify who is there and what they are doing.

#### Theme E: Design procedure

**Figure 8.**
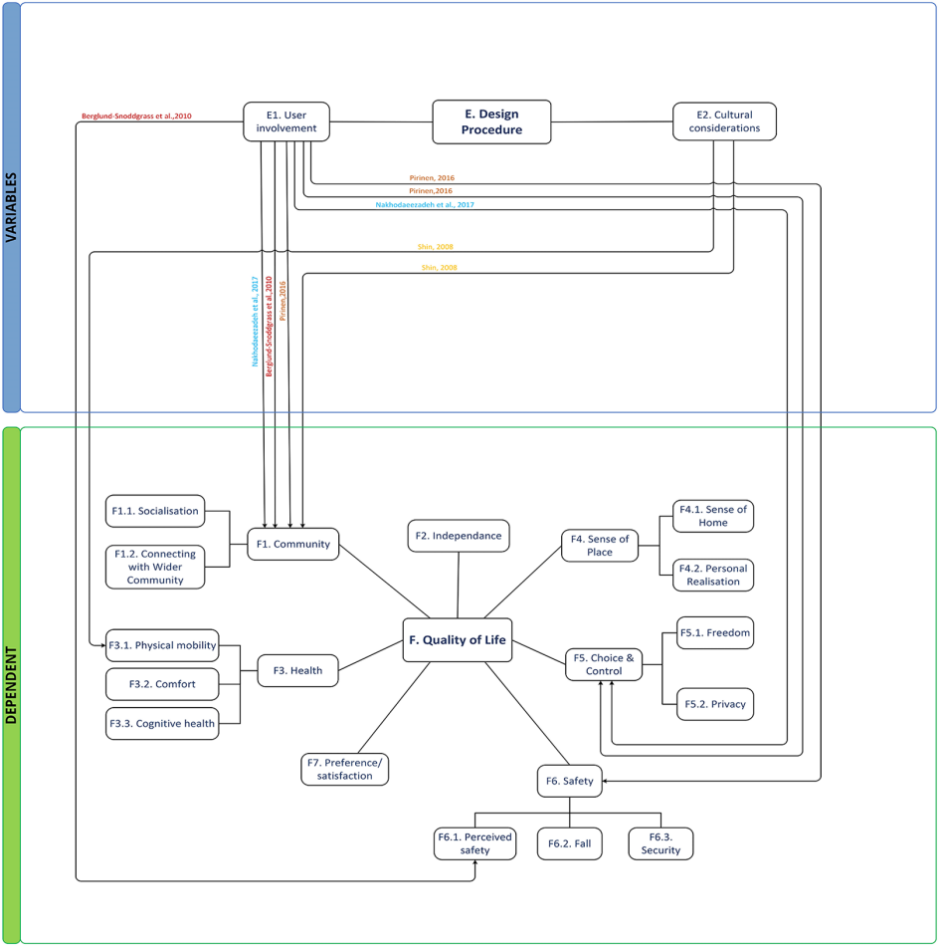
Thematic conceptual diagram between Group E and dependent groups.

There were two different subthemes discussed: *user involvement* and *cultural consideration* (Figure 8). Pirinen (2016) discussed the discrepancies between housing concepts developed by versus housing concepts for older people. Including older people in the design process raised elders from subjects to main agents and design resources. While beyond the scope of the specific study, differences in the designs produced by the direct users vis-à-vis conventional design approaches appear meaningful and worthy of further discussion. Nakhodaeezadeh et al. (2017) examines the interaction between QOL and socio-physical environment and local culture in Iranian elders, in which having a guest room reinforces the social support network of older people.

#### Theme F: Quality of life (QOL)

*Community* is a subtheme connected to QOL for older people in their home environment. Socialization and social support from their community were frequently mentioned by users in several studies (Berglund-Snodgrass & Nord, 2019; Burton & Sheehan, 2010; Gobbens & van Assen, 2018; Lindahl et al., 2018; Nakhodaeezadeh et al., 2017; Orrell et al., 2013; Shin, 2018). Connection with the wider community was found to be low, whereas socialization within the community was high (Orrell et al., 2013). *Independence autonomy* is supported by the physical environment by highlighting that care needs were reduced after house modification (Carnemolla & Bridge, 2014, 2019)*. Independence* is a major goal of housing design for older people (Kneale & Smith, 2013). *Health*-related QOL (health and well-being) is linked to physical mobility, comfort, and cognitive ability (Carnemolla & Bridge, 2019; Currin et al., 2012; Kim & Portillo, 2018; Lindahl et al., 2018; Nakhodaeezadeh et al., 2017; Orrell et al., 2013). Smith et al. (2016) measured outdoor time and concluded that community accessibility led to increased mobility. *Sense of place* is highly correlated with location and spatial flexibility (Berglund-Snodgrass & Nord, 2019; Orrell et al., 2013) and is interpreted as *sense of home* and *personal realization*, which pertains to a sense of belonging and adapting to interactions with new context (Orrell et al., 2013). *Sense of home* is understood in relation to size, space configuration, and accessibility (Orrell et al., 2013; Van Steenwinkel et al., 2017). However, for people with higher dependency, functionality was more appreciated than a home-like design (Currin et al., 2012), although accessibility adversely affected home-likeness (Orrell et al., 2013). *Choice and control* encompass the concept of privacy and feelings of freedom, which are correlated with socialization and a belief of environmental control. Choice of space is important owing to the diverse needs of individuals (Burton & Sheehan, 2010). Chin and Quine (2012) suggest that increasing opportunities for choice and control could result in an improved sense of self and improved QOL. Control can be assessed with the HCB Questionnaire (Kylén et al., 2014). Residents in ECH reported more objective control than residents in home care settings (Lindahl et al., 2018). Freedom was described to be associated with walkability in the immediate environment (Rodiek & Fried, 2005), visual accessibility, and generous size (Van Steenwinkel et al., 2017). Feelings of privacy were supported by spatial flexibility and layout, home-likeness, variety, and choice of spaces (Burton & Sheehan, 2010; Chin & Quine, 2012). *Satisfaction* represents an endorsement of a positive attitude toward one’s life and is associated with greenery, attractiveness of the building, environmental comfort, and outlook (Burton & Sheehan, 2010; Rodiek & Fried, 2005). The subtheme *safety* is categorized into *perceived safety*, *fall hazard*, and *security*. *Perceived safety* relates to how safe the residents feel about their physical environment. Accessibility in a social or material context that supports aging, and spatiality where a strong cohesion naturally occurs, influenced residents’ perception of safety (Berglund-Snodgrass & Nord, 2019). In addition, building design elements such as grabrails, which even if not presently used, affected future functional change (Lindahl et al., 2018). Environmental *fall hazard* was affected by poor accessibility, which increases fall risk (i.e., surface height, width, and slippery textures in the bathroom and kitchen). Insufficient space to maneuver and pass, inappropriate furniture design, lack of handrails, and lighting also affected fall rate (Kim & Portillo, 2018). *Security* involves concerns for social context and the surrounding neighborhood (Carnemolla & Bridge, 2014; Gobbens & van Assen, 2018; Lindahl et al., 2018; Orrell et al., 2013; Shin, 2018). Security is a subtheme which positively predicts residents’ QOL in ECH (Orrell et al., 2013). Clean environment, limited noise, low-crime areas, and monitored access are reported to foster a sense of safety and security (Shin, 2018).

### Thematic Framework

Figure 3 represents different subthemes in each theme. Using evidence from the literature, we have established a framework via the connections between themes and subthemes with Theme F. Quality of Life. Repeated relationships between physical elements and user experience are accessibility-independence, hand-rail-independence, communal space-socialization, modification-independence, and walkability-physical activity. Except for these elements, all other relationships were noted to be single instances.

### Design Features

The next step for designers and architects is to incorporate these findings into future building designs and to ensure that the core themes are considered to inform new HWC plans. We have identified several examples of design features for consideration to ensure that HWC schemes meet the needs of residents under the five overarching themes (see Table 4). This list is not exhaustive, and further work may be warranted to ensure that HWC schemes can be designed with the aid of a comprehensive checklist of design features to be considered by architects.

**Table 4.** Examples of Design Features for Each Theme.

Theme	Examples of Design Features That Architects Could Consider
A	Double sliding partition walls increase spatial flexibility in independent housing schemes. They allow to extend the living room (or any closed space), for example, if the residents entertain guests or if their care needs expand to require more space or other similar scenarios. Architects can minimize obscurity (increase lighting level) of the space through appropriate layout design or via (architectural elements) such as double-height windows.
B	Dedicated storage space for mobile aid near points of transfer (along with handrail) may allow to reduce the environmental hazard of fall. In terms of flooring for wet rooms or walk-in showers, matte-finish mosaic tile or cork flooring are good options for relatively independent older adults as they are nonslippery materials in addition to being moderately wheelchair friendly.
C	Weight and height of windows designed in consideration of users’ capacity, low cabinets, and cupboards can encourage users’ independent daily activities and perceived safety.
D	While the impact of a green view is well known, it would be ideal to also have double-side views, as they might encourage a sense of connectedness to the world.
E	Codesign is an approach to the design process involving the residents’ active participation, which naturally reflects cultural considerations. Users who participated in building concepts of their communal residential setting presented a greater sense of community, satisfaction with the facilities, and autonomy.

## Discussion

The purpose of this scoping review and thematic analysis was to synthesize the research on HWC design to develop a framework for designers to create a novel ECH typology. To address the need for objective data regarding physical design elements, we created thematic maps to elucidate the elements of the physical environment that contribute to the subjective dimensions of QOL. This resulted in a framework for the design of HWC that maps the diverse needs of the older adult onto the various effects of their physical environment.

There are numerous tools for objectively assessing the living environment. These tools are single score assessments that link the built environment and health or attribute a single score to the built environment. Here, we identified the following predesigned tools: SCEAM (2004), EVOLVE (2010), and HOUSING ENABLERS (1979). However, caution should be used in using these tools as they may not consider the various levels and types of care for older people—who clearly need to be integrated into the physical environment. To develop scientific evidence, more data need to be accumulated that encompasses the interrelationships between building elements and corresponding improvements to functionality and QOL. This scoping review creates a road map of existing evidence in housing design for older people, while simultaneously addressing the need to integrate built environment with care provision. Research on care programs or regimes also needs to be studied in qualitative, quantitative, and mixed methods studies.

***To develop scientific evidence, more data need to be accumulated that encompasses the interrelationships between building elements and corresponding improvements to functionality and QOL.***

Methodological concerns arise in studies involving housing for older people (Cutler, 2007) such as multiplicity of variables in relevant research and a lack of consensus for which variables are most important for both users and service providers. This is compounded by a lack of consistent definitions or units of measure across the extant literature. Having no criteria by which to formally evaluate housing appropriateness and satisfaction hinders the integration of complex variables to establish a causal link between environment and QOL. The majority of evidence is from qualitative studies such as interviews and cross-sectional studies with QOL variables (Cutler, 2007). Tools such as those developed by Kylén et al. (2014) assess environment and QOL allow for the conversion of data and uniting of outcomes of different studies.

***This is compounded by a lack of consistent definitions or units of measure across the extant literature.***

***The majority of evidence is from qualitative studies such as interviews and cross-sectional studies with QOL variables***

Our review yielded only one longitudinal study, while three studies used standardized tools. Ten studies used subjective measures. Additionally, most research targets long-term dwellers, which could be considered beneficial to establish the duration of potential interactions between the housing environment and QOL over time. On the other hand, it could lead to a reduced ability to detect issues in cases where subjective variables are used, as the subjects may become accustomed to them, thereby skewing the interactions.

### Strengths and Limitations

The present study represents the first attempt to create a framework to inform the design of HWC based on a comprehensive scoping review that links specific design elements to QOL outcomes using thematic analysis mapping. The present study also overcame common methodological barriers to assessing the effects of the physical environment to QOL by integrating subjective assessment and objective measurement. This evidence-based approach to assessing HWC was based on a global selection of studies for which quality was assessed and reports data that are transparent and objective. The results were synthesized to provide a new and original piece of work that will be a foundation for future research. This is an innovative attempt to use established and scientifically robust methods to review and link evidence in a discipline that is new to this type of analysis and will improve the quality of future studies in this area.

There are several limitations of the present study. First, owing to the lack of studies on the topic of HWC, a broader set of papers was collected, including studies focused on living environments similar to HWC, as HWC is deemed to cover ECH settings. Second, even though the quality assessments in the present study were performed by modifying validated quality assessment tools, these tools are typically focused on healthcare research, and thus their application to the research question may not be directly applicable. Third, owing to limited resources and time, the project was not registered as a systematic review a priori. Fourthly, in designing literature searches for a novel topic, there are difficulties in setting the search parameters in order to cover the full scope of the study and as such there may be relevant publications that might not be indexed in databases using conventional terms. Therefore, defining appropriately specific search parameters might not ensure the capture of all related studies. Furthermore, while this review was inclusive with regard to geographical contexts, the restriction to English language may have contributed to a predominance of studies from Western countries. A future review could establish whether similar studies published in languages other than English elucidate further data not covered by our thematic framework. However, there are likely challenges for future researchers in comparing HWC-related parameters between contexts with very different cultural and contextual prerequisites.

Lastly, establishing absolute causal relationships between the physical environment and QOL, including clinical health outcomes, is difficult. More research is needed to both expand on the initial framework created in the present study and to establish methods to strengthen the connections between the physical environment and QOL. Nevertheless, the present study consulted and adhered to best practice guidelines and provides a road map for both researchers interested in HWC as a model and for designers of HWC schemes.

## Conclusion

The evidence reviewed in this article provides a conceptual framework for how the physical elements of housing environment impact QOL, especially within specific contexts. The originality of the study lies in the knowledge gap at the intersection of the HWC model, the physical environment, and QOL for older people. There is a considerable lack of research on the array of architectural design elements for this new housing typology for older people, as well as the resulting impacts on QOL, and a clear need for further investigation to elucidate this relationship. This suggests that when designers and architects conceptualize and design, they should consider cross-examining the outcomes of the studies from this review. The present work could serve as a basis for the development of a consensus on a uniform framework for designed schemes. In this sense, it is hoped that the thematic framework identified in this review serves not only as a basis for further research for HWC schemes but can also be helpful for designers and architects to implement in practice hereafter. Moreover, design themes that have not been reported extensively (e.g., design space for assistive technology, maneuverability, and visibility) could be further verified with end users so that the validity of the design themes can be established across different contexts. This examination should use qualitative measures such as interviewing residents and stakeholders, both before and after moving into HWC schemes. Additionally, involving the target population in the design process can raise older people from subject to main design resources. Such a design process can be facilitated by developing reliable and validated tools to accurately capture QOL in response to the built environment for older people. Accordingly, a framework that encompasses several different themes and levels of evidence should be established via the integration of subjective assessments from residents and objective measurement through caregivers and/or support workers. An ecological evidence-based design framework can be established via the rigorous design processes of designers and architects to further optimize the physical environment and maximize QOL in the aging population.

## Implication for Practice

- This review proposed a practical method using scoping review and thematic analysis mapping to comprehensively search for synthesized evidence that links the design of the physical environment with assessment of QOL.
- This research established a framework that provides a useful, comprehensive, and evidence-based summary for designers and architects and pinpoints the key design areas that contribute to older people’s QOL.
- This review highlights that architecture for older—which are likely to impact quality of life—people should consider multiple factors that may not have previously been considered by designers—beyond accessibility. In addition, designers can make decisions from own assessing the validity of evidence from research.
- A design method that involves end users, such as codesign, warrants more attention in designing housing with care. In a modern society—especially where diversity and inclusiveness are required—design for culture-specific cohort is noteworthy, most notably how design can help integration of different cohorts in a community.

## Supplemental Material

Supplemental Material, sj-pdf-1-her-10.1177_19375867221113359 - Housing With Care for Older People: A Scoping Review Using the CASP Assessment Tool to Inform Optimal Design Click here for additional data file. Supplemental Material, sj-pdf-1-her-10.1177_19375867221113359 for Housing With Care for Older People: A Scoping Review Using the CASP Assessment Tool to Inform Optimal Design by Suyee Jung, Lesley Uttley and Junjie Huang in HERD: Health Environments Research & Design Journal
