# Supplementary material for: Housing With Care for Older People: A Scoping Review Using the CASP Assessment Tool to Inform Optimal Design
Source: HERD. 2022 Aug 22;15(4):299–322. doi: 10.1177/19375867221113359 (PMC9523824; doi:10.1177/19375867221113359)
Supplement: Supplemental Material, sj-pdf-1-her-10.1177_19375867221113359 - Housing With Care for Older People: A Scoping Review Using the CASP Assessment Tool to Inform Optimal Design [file sj-pdf-1-her-10.1177_19375867221113359.pdf]

Appendix 1. Search strategy on Medline

Key concept	Search entry terms	Medical Subject Headings (MeSH)	Sub-headings
Key concept 1. Elderly	Elderly	Aged	
	Older people	Aged	
	Older adults	Aged	
	Senior	Aged	
	Aged 65	Aged	
	Ag#ing	Aged	
Key concept 2. Housing	Housing	Housing	Housing for elderly
	Housing with care	Housing	Homes for aged
	Extra care housing		
	Residen*		
	Home		
	House		
	Dwell*		
	Living environment	Housing	Independent living
Key concept 3. Design	Design	Environment design	Architectural accessibility<br>Interior design and furnishings<br>Floor and floorcoverings
	Cost		
	Quality of life	Quality of life	
	Well#being		
	Stay*		
	Safety		
	Independen*	Independent living	Activities of daily living

Appendix 2. Adapted CASP for qualitative research.

1. Was there a clear statement of the aims of the research?

2. Is qualitative methodology appropriate?

3. Was the research design appropriate to address the aims of the research?

4. Was the recruitment strategy appropriate to the aims of the research?

5. Was the data collected in a way that addressed the research issue?

6. Has the relationship between researcher and participants been adequately considered?

7. Have ethical issues been taken into consideration?

8. Was the data analysis sufficiently rigorous?

9. Is there a clear statement of findings?

10. How valuable is the research?

11. Demographical applicability

12.Is the focus on architectural design element?

13. Is the result applicable to the design of 'Housing with care'?

Appendix 3. Adapted CASP for cohort study

1. Did the study address a clearly focused issue?

2. Was the cohort recruited in an acceptable way?

3. Was the exposure accurately measured to minimise bias?

4. Was the outcome accurately measured to minimise bias?

5-a. Have the authors identified all important confounding factors?

5-b. Have they taken account of the confounding factors in the design and/or analysis?

6-a. Was the follow up of subjects complete enough?

6-b. Was the follow up of subjects long enough?

7. What are the results of this study?

8. How precise are the results?

9. Do you believe the results?

10. Can the results be applied to the local population?

11. Do the results of this study fit with other available evidence?

12. What are the implications of this study for practice?

13. Is the result demographically applicability to housing with care?

14. Is the focus on architectural element?

15. Is the result applicable to the design of 'Housing with care'?

Appendix 4. Adapted CASP for case control research

1. Did the study address a clear focused issue?

2. Did the authors use an appropriate method to answer their question?

3. Were the cases recruited in an acceptable way?

4. Were the controls selected in an acceptable way?

5. Was the exposure accurately measured to minimise bias?

6-a. Aside from the experimental intervention, were the groups treated equally?

6-b. Have the authors taken account of the potential confounding factors in the design and/or in their analysis?

7. How large was the treatment effect?

8. How precise was the estimate of the treatment effect?

9. Do you believe the results?

10. Can the results be applied to the local population?

11. Do the results of this study fit with other available evidence?

12. Is the result demographically applicability to housing with care?

13. Is the focus on architectural design element?

14. Is the result applicable to the design of 'Housing with care'?
